# Flow cytometry and growth‐based analysis of the effects of fruit sanitation on the physiology of *Escherichia coli* in orange juice

**DOI:** 10.1002/fsn3.947

**Published:** 2019-02-07

**Authors:** Amir H. P. Anvarian, Madeleine P. Smith, Tim W. Overton

**Affiliations:** ^1^ Bioengineering School of Chemical Engineering The University of Birmingham Birmingham UK; ^2^ Institute of Microbiology & Infection The University of Birmingham Birmingham UK; ^3^ Present address: University of Lincoln National Centre for Food Manufacturing Holbeach Technology Park, Holbeach Lincolnshire UK

**Keywords:** *Escherichia coli*, flow cytometry, fruit sanitation, orange juice, VBNC cells

## Abstract

Chlorine‐based solutions are commonly used to sanitize orange fruits prior to juice extraction. We used flow cytometry (FCM) to investigate the physiology of *Escherichia coli* following its subjection to chlorine‐based solutions and alternative sanitizing agents (H_2_O_2_ and organic acids). Green fluorescent protein (GFP)‐generating *E. coli* K‐12 were washed with 50–200 ppm available chlorine (AC), 1%–5% H_2_O_2_, 2%–4% citric acid, 4% acetic acid, or 4% lactic acid, after which they were added to 1.2 μm‐filtered orange juice (OJ). Cell physiology was investigated with FCM during storage at 4°C, and culturability was determined using plate counting. Analysis of GFP fluorescence allowed estimation of intracellular pH (pH_*i*_). FCM results demonstrated an inverse relationship between the concentration of AC or H_2_O_2_ and cellular health in OJ. Higher concentrations of sanitizer also resulted in a significantly greater number of viable but nonculturable (VBNC) cells. Real‐time FCM showed that supplementation of AC with 2% citric acid, but not with 100 ppm of Tween‐80, led to a significant reduction in pH_*i*_ of the cells incubated in OJ, and that the majority of the reduction in pH_*i*_ occurred during the first 2 min of incubation in OJ. Organic acids were found to be more effective than both AC and H_2_O_2_ in reducing the pH_*i*_, viability, and culturability of the cells in OJ. The results confirmed the hypothesis that consecutive subjection of *E. coli* to maximum legally permitted concentrations of sanitizers and OJ induces the VBNC state. Furthermore, we demonstrate successful application of FCM for monitoring the efficacy of washing procedures.

## 1 INTRODUCTION

Production of unpasteurized orange juice (OJ) consists of many stages such as harvest, washing, extraction, packaging, and storage. From a food safety point of view, the most important critical control point (CCP) during the production of freshly squeezed OJ is the sanitation step (Schmidt, Sims, & Ismail, 1997) which is required by the FDA to provide 5‐log pathogen reduction (FDA, 2004). This is mainly because, subsequent to orange fruit sanitation, there is no further step such as pasteurization to eliminate the potential pathogens. Acidic fruit juices have been implicated in outbreaks of highly acid‐resistant *Escherichia coli* O157:H7; hence, effective sanitation of fruit surfaces is crucial for reducing the risks posed by this pathogen, especially as. *E. coli* O157:H7 has been shown to survive in OJ (Eblen et al., 2004).

Available chlorine (AC) is the most frequently used sanitizer in the food industry and is used for the purpose of washing the surface of various fruits, processing work surfaces, and decontaminating the washing water. AC concentrations of 50–200 ppm with a contact time of 1–2 min are commonly used for washing fresh produce including oranges (Parish et al., 2003; Suslow, 2000). Consequently, numerous studies have investigated the effectiveness of AC for eliminating *E. coli* from the surface of orange fruits (Bagci & Temiz, 2011; Martinez‐Gonzales, Martinez‐Chavez, Martinez‐Cardenas, & Castillo, 2011; Pao & Davis, 1999; Pao, Davis, & Kelsey, 2000). However, these studies have only measured elimination of bacteria using plate counts; the physiological state of the bacteria has not been determined using nongrowth‐based methods. Considering that an AC concentration as low as 0.4 ppm can cause sublethal injury and induce a viable but nonculturable (VBNC) state in *E. coli* whereby bacteria are viable but do not grow on agar plates (Kolling & Matthews, 2001; Singh, Yeager, & McFeters, 1986), and measurement of viability using plate counts could grossly underestimate the number of viable bacteria. VBNC *E. coli* including AC‐injured bacteria have also been previously shown to be capable of revival, growth, and pathogenicity in vivo when the stress conditions were removed (Palmer, Baya, Grimes, & Colwell, 1984; Pao, Davis, Kelsey, & Petracek, 1999).

In addition to AC, H_2_O_2_ and organic acids such as citric, lactic, and acetic acids have also been suggested as suitable alternatives to AC for sanitation of fresh produce (Parish et al., 2003). However, H_2_O_2_, lactic acid, and acetic acid are also known to induce the VBNC state in *E. coli* (Li, Ahn, & Mustapha, 2005; Oliver, 2005). *E. coli* K‐12 strain SSC1 used here constitutively expresses green fluorescent protein (GFP) (Miao, Ratnasingam, Pu, Desai, & Sze, 2009); It has previously been demonstrated that decreases in intracellular pH (pH_*i*_) result in GFP deactivation (Kneen, Farinas, Li, & Verkman, 1998). The primary aim of this study was to use flow cytometry (FCM) to investigate the effects of washing *E. coli* K‐12 SCC1 with the sanitizers AC, H_2_O_2_, and organic acids on the viability, physiological state, and pH_*i*_ of the cells as well as their culturability as measured using plate counts, before and after their inoculation in OJ. *E. coli* K‐12 has previously been used as a model for *E. coli* O157:H7 (Anvarian, Smith, & Overton, 2016; Valdramidis, Geeraerd, & Van Impe, 2007). We reveal the presence of VBNC bacteria in OJ following washing with both AC and H_2_O_2_, use real‐time FCM to determine the effects of acid and surfactant on available chlorine washing, and demonstrate the effects of organic acids on cells subsequently incubated in OJ.

## 2 MATERIALS AND METHODS

### 2.1 Bacterial strain and preparation of the culture

*Escherichia coli* K‐12 SCC1 (MG1655 P_A1/04/03_‐*gfp*mut3*) (Miao et al., 2009) was used in this study. *Escherichia coli* were taken from frozen glycerol stocks, grown for 24 hr on a nutrient agar plate at 37°C, then restreaked and grown for a further 24 hr at 37°C. A single colony of *E. coli* was inoculated in 20 ml of 2× LB (Lysogeny broth; 20 g/L tryptone, 10 g/L yeast extract (both Difco) and 10 g/L NaCl (Sigma, UK)) and allowed to grow in a shaking incubator (New Brunswick Scientific Innova 4000) for 18 hr at 37°C with agitation (150 rpm). The overnight culture was subsequently diluted 1:1,000 in 50 ml of 2× LB medium and grown for another 24 hr under the same conditions to obtain late‐stationary‐phase culture.

### 2.2 Orange juice

Orange juice was obtained from a local retailer and centrifuged at 17,696 *g* for 40 min (Beckman J2‐21, Beckman Coulter) to remove pulp. The pulp‐free supernatant was then filtered through a sterile filter paper of 1.2 μm pore size (Whatman, Maidstone, UK) to prevent the interference of OJ cloud particles with bacterial detection using FCM, as previously described (Anvarian, Smith, & Overton, 2018; Anvarian et al., 2016).

### 2.3 Sanitizing solutions

Sodium hypochlorite (NaOCl; Sigma) containing 10%–15% AC (Sigma) was utilized for preparing chlorine‐based sanitizers. The estimated midrange value of 12.5% AC was used for calculating the concentration of NaOCl needed for preparation of the sanitizers. The AC‐containing solutions (50, 100, 200, and 250 ppm) were prepared by diluting NaOCl in distilled water (dH_2_O) just before use. In order to prepare the acid‐supplemented AC (ASAC) and surfactant‐supplemented AC (SSAC) solutions, citric acid (BDH Ltd., UK) or Tween‐80 (Sigma) was used as acidulant and surfactant, respectively. With regard to ASAC (200 ppm AC + 2%, i.e., 20,000 ppm, citric acid), the solution with 250 ppm AC was gently added to a 10% (w/v) citric acid solution in the ratio of 4:1 (v/v). For SSAC, 250 ppm AC solution was mixed with 500 ppm Tween‐80 in the ratio of 4:1 (v/v) in order to achieve a solution containing 200 ppm AC and 100 ppm Tween‐80. A 30% (w/w) solution of H_2_O_2_ (Sigma) in H_2_O (density of 1.11 g/ml) was used for preparing 1%, 2.5%, or 5% (all w/w) H_2_O_2_ sanitizing solutions by diluting the 30% H_2_O_2_ in sterile dH_2_O in the ratio of 1:32.19, 1:12.21, and 1:5.55, respectively. AC and H_2_O_2_ solutions were prepared just before use. Citric acid solution of 2% and 4% (w/v; 2CA and 4CA, respectively) was prepared by dissolving citric acid in dH_2_O. Lactic acid solution (85% (w/w) in H_2_O; Sigma, density 1.206 g/ml) was diluted in dH_2_O in the ratio of 1:24.42 in order to prepare a 4% (w/w) solution (4LA). The 4% (w/v) acetic acid solution (4AA) was prepared by diluting glacial acetic acid (Fisher Scientific, density: 1.049 g/ml) in dH_2_O. All organic acid solutions were prepared on the day of the experiment and filter‐sterilized before use.

### 2.4 Washing procedure

Two ml of the culture was transferred to a 2.5 ml centrifuge tube (Sarstedt, UK). Cells were harvested by centrifugation (5 min at 16,873 *g*), the supernatant was disposed, and the pellet was dispersed in 50 μl of sterile Dulbecco's Phosphate‐Buffered Saline (PBS). Next, 1 ml of sanitizer at 30°C prepared 10 min prior to the experiment was added to the cell suspension and gently mixed for 2 min by rotating the tubes in a test tube rotator (20 rpm). This temperature (30°C) was chosen in an attempt to mimic the recommended temperature for a sanitizer during the washing stage of the fruits with core temperature of around 22.5°C (room temperature). The positive temperature difference has been shown to prevent infiltration of pathogens into the fruit pulp (Zhuang, Beuchat, & Angulo, 1995). After washing, cells were harvested by centrifugation (16,873 g), washed with PBS, and dispersed in 1 ml of fresh PBS. Subsequently, 3 × 10^9^ cells were added to 15 ml of filtered OJ in glass universal bottles. Samples were then incubated at 4°C for 13 days with no shaking.

### 2.5 Flow cytometry and plate counting

At each time point, samples were diluted in PBS, stained with PI (Sigma) and Bis‐(1,3‐Dibutylbarbituric Acid) Trimethine Oxonol (DiBAC_4_(3) or BOX; Life Technologies), and analyzed with a BD Accuri C6 flow cytometer (BD, Oxford, UK). The stock solution of PI was prepared by dissolving PI in dH_2_O at concentration of 300.12 μM. The stock solution of BOX was prepared by first dissolving the BOX in dimethyl sulfoxide (19.36 mM) followed by dilution in PBS in the ratio of 1:1,000 (19.36 μM). BOX was added to the samples at final concentration of 191.64 nM. EDTA was also added at a final concentration of 400 μM in order to facilitate staining with BOX. In total, 20,000 events were recorded per sample. Fluorescence was detected using 533/30 bandpass and 670 longpass filters corresponding to GFP/BOX and PI fluorescence, respectively. Data were analyzed using CFlow (BD). In addition, samples were serially decimally diluted in maximum recovery diluent (MRD; 8.5 g/L NaCl, 1 g/L peptone) and plated on nutrient agar plates (Oxoid), which were incubated at 37°C for 48 hr in order to determine the aerobic plate count. Throughout, *t* test analysis using Microsoft Excel was used, with a significant *p* value of 0.05. Error bars shown on figures are the ±*SD* of the mean value obtained at each data point.

## 3 RESULTS

### 3.1 Effect of available chlorine concentration on physiology and viability

**Figure 1:**
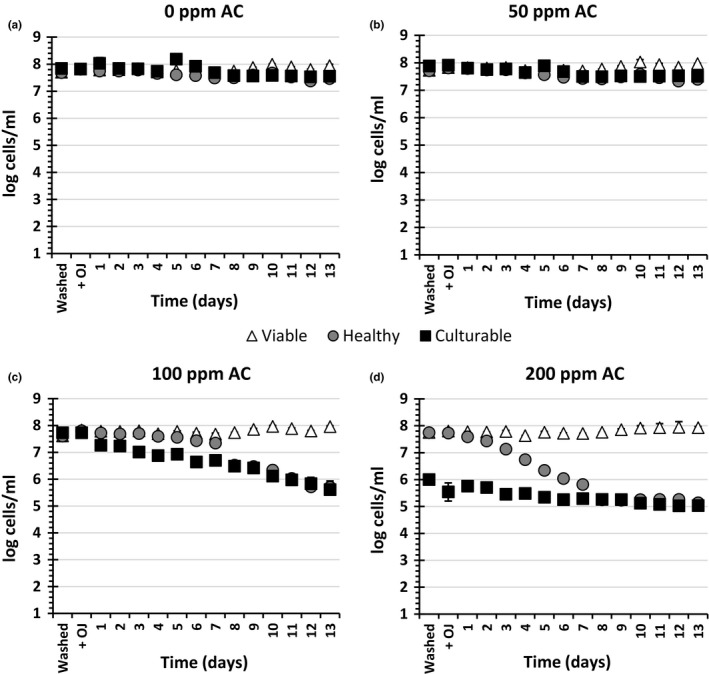
The effects of washing stationary‐phase *Escherichia coli* K‐12 SCC1 with different concentrations of available chlorine (AC) on viability, health, and culturability during incubation in orange juice (OJ) at 4°C. Stationary‐phase *E. coli* were washed with (a) dH_2_O (0 ppm AC), (b) 50 ppm AC, (c) 100 ppm AC, or (d) 200 ppm AC for 2 min. Cells were washed with Phosphate‐Buffered Saline (PBS) and 3 × 10^9^ cells dispersed in 15 ml of OJ (filtered with 1.2 μm filter paper). Samples were then incubated at 4°C for 13 days. At each time point, samples were diluted in PBS, stained with PI, and analyzed by flow cytometry, and aerobic viable count was determined. The experiment was repeated twice each with a duplicate. The reported values are the mean values of duplicate samples for a representative experiment. “Washed” refers to cells postwashing with AC but before addition of OJ, while “+OJ” refers to time 0 hr postaddition of OJ

Figure 1 shows the effects of washing cells with dH_2_O (0 ppm AC) or AC solutions (50–200 ppm) on the viability and physiological state of the cells, immediately after the washing step (Washed) or after addition of OJ (+OJ) and during their incubation at 4°C (to simulate refrigerated storage) for 13 days. The OJ was clarified to permit the use of FCM without interference from the cloud particles; the effect of OJ clarification on the physiology and viability of *E. coli* K‐12 has previously been described (Anvarian et al., 2016). Cells that retained GFP fluorescence and did not stain with PI (which stains dead cells red) were considered healthy with near neutral pH_*i*_ (GFP^+^/PI^−^) while GFP^−^/PI^−^ cells were considered to be stressed but viable bacteria with a pH_*i*_ of <5 (Anvarian et al., 2016; Kneen et al., 1998). Viable bacteria numbers reflect all PI^−^ cells; culturable bacteria were determined by plate counts. In the case of the control samples, immediately after addition of OJ (+OJ), more than 99% of the cells were GFP^+^ (healthy). Washing cells with 50 ppm AC did not affect the overall number of viable or culturable bacteria throughout the experiment. On the other hand, washing with 100 ppm or 200 ppm AC had a highly significant effect on numbers of both viable and culturable cells. In the case of 100 ppm AC, at time 0 hr, no difference was observed between numbers of viable and culturable cells before and after addition of OJ. However, compared to control samples, there was a highly significant decrease in culturable cells during the next 13 days of the study and in numbers of healthy cells between day 7 and day 13 (both *p *<* *0.0001).

Unlike the other three conditions, there was a highly significant reduction in culturable cells immediately after washing with 200 ppm AC (Washed; *p *<* *0.0001); however, the decrease in healthy cells as determined by FCM was far slower, suggesting formation of a VBNC population (Li, Mendis, Trigui, Oliver, & Faucher, 2014). After 8 days, the number of healthy cells had declined to match numbers of culturable cells. By day 13, the level of reduction in culturable cells was similar to that observed for cells washed with 100 ppm AC. Nonetheless, cells that remained culturable after the initial treatment with 200 ppm AC were more successful in retaining their culturability than those washed with 100 ppm AC, indicating a possible induction of resistance to components of OJ.

### 3.2 Effect of hydrogen peroxide

**Figure 2:**
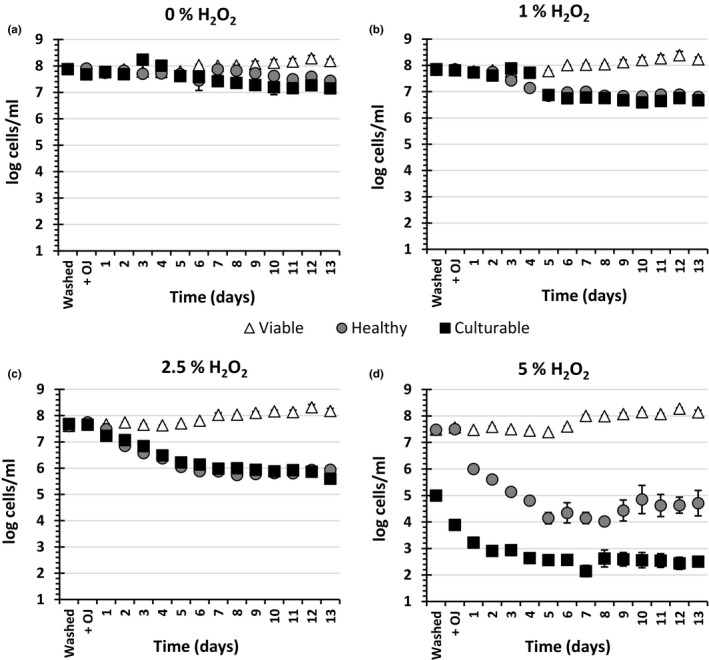
The effects of washing stationary‐phase *Escherichia coli* K‐12 SCC1 with different concentrations of H_2_O_2_ on viability, health, and culturability during incubation in orange juice (OJ) at 4°C. The method was similar to that described in Figure 1; however, instead of using available chlorine, cells were washed with (a) dH2O (0% H_2_O_2_), (b) 1% H_2_O_2_, (c) 2.5% H_2_O_2_, or (d) 5% H_2_O_2_ before inoculation in filtered OJ

Compared to AC, H_2_O_2_ has been suggested as a more effective sanitizer against *E. coli* (Sapers, Miller, & Mattrazzo, 1999). Therefore, it was decided to study the effects of washing with H_2_O_2_ on the physiology of *E. coli* before and after addition of OJ. No significant difference was observed between numbers of culturable cells washed with dH_2_O, 1% H_2_O_2_, or 2.5% H_2_O_2_, either after washing or after addition of OJ (Washed and +OJ, respectively) (Figure 2). However, 5% H_2_O_2_ was found to be significantly more effective than 200 ppm AC (Figure 1) in reducing the culturability of the cells (*p *<* *0.01). As with the AC experiment (Figure 1), culturability of cells treated with 5% H_2_O_2_ declined far faster than the number of healthy cells determined by FCM, indicating a VBNC phenotype, whereas lower concentrations of H_2_O_2_ resulted in similar numbers of culturable and healthy cells. There was an inverse dose‐dependent reduction in numbers of both culturable and healthy H_2_O_2_‐washed cells incubated in OJ. Compared to dH_2_O‐washed cells, the post‐OJ‐incubation reduction in culturable and healthy cell counts during the course of the study was significantly higher when cells were washed with 1% H_2_O_2_ (*p *<* *0.05), 2.5% H_2_O_2_ (*p *<* *0.01), and 5% H_2_O_2_ (*p *<* *0.05), respectively.

### 3.3 Acid and surfactant supplementation of AC

**Figure 3:**
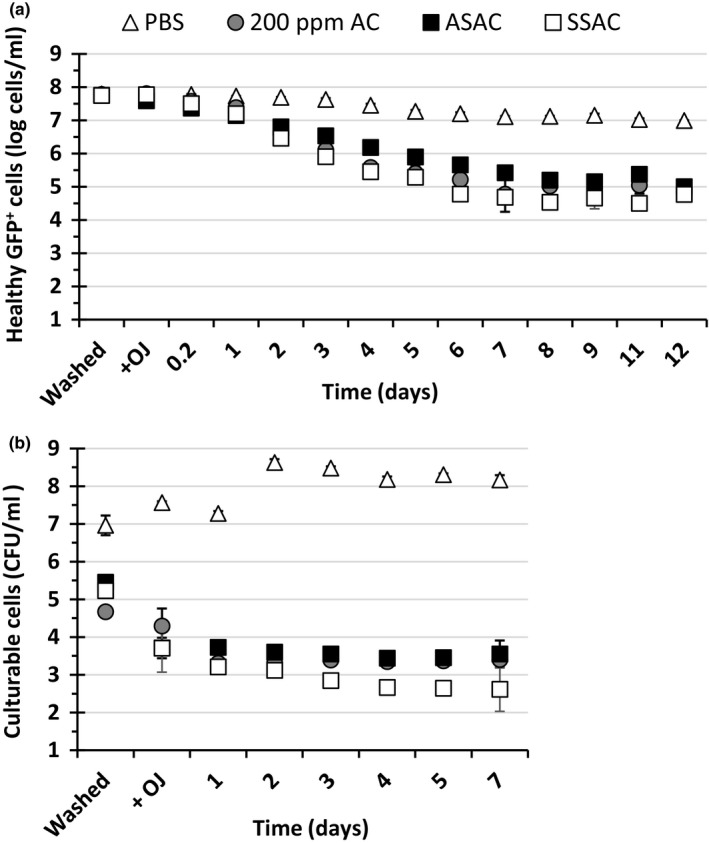
The effect of washing *Escherichia coli* K‐12 SCC1 with Phosphate‐Buffered Saline (PBS), available chlorine (AC), ASAC, and SSAC on health and culturability during incubation in orange juice (OJ) during 12‐day incubation at 4°C. The method used for this experiment was similar that described in Figure 1 except cells were washed with PBS (control), 200 ppm AC (AC), acid‐supplemented AC (200 ppm AC + 2% citric acid; ASAC), or surfactant‐supplemented AC (200 ppm AC + 100 ppm Tween‐80 (SSAC). (a) The total log healthy (GFP ^+^/PI ^−^) cells/ml as determined by flow cytometry. (b) Total culturable cells as determined by plate counting

Numerous studies have demonstrated improved antimicrobial efficacy of AC when adjusted to mild acidic pH of approximately 6–6.5 or supplemented with surfactants (Adams, Hartley, & Cox, 1989; Martinez‐Gonzales et al., 2011; Sapers, Miller, Annous, & Burke, 2002). In this study, 200 ppm AC was supplemented with either 2% citric acid (acid‐supplemented AC; ASAC) or 100 ppm Tween‐80 (surfactant‐supplemented AC; SSAC). Figure 3a shows the effects of supplementation of AC solution with acid or surfactant on the number of healthy after washing and after addition of OJ. After 1 day, compared to samples containing cells washed with AC or SSAC, the number of healthy cells in samples containing ASAC‐washed cells was significantly higher (*p *<* *0.05). With regard to SSAC samples, the mean log number of healthy cells was slightly lower than those found in AC‐washed samples, although the difference between the two conditions was not statistically significant.

**Figure 4:**
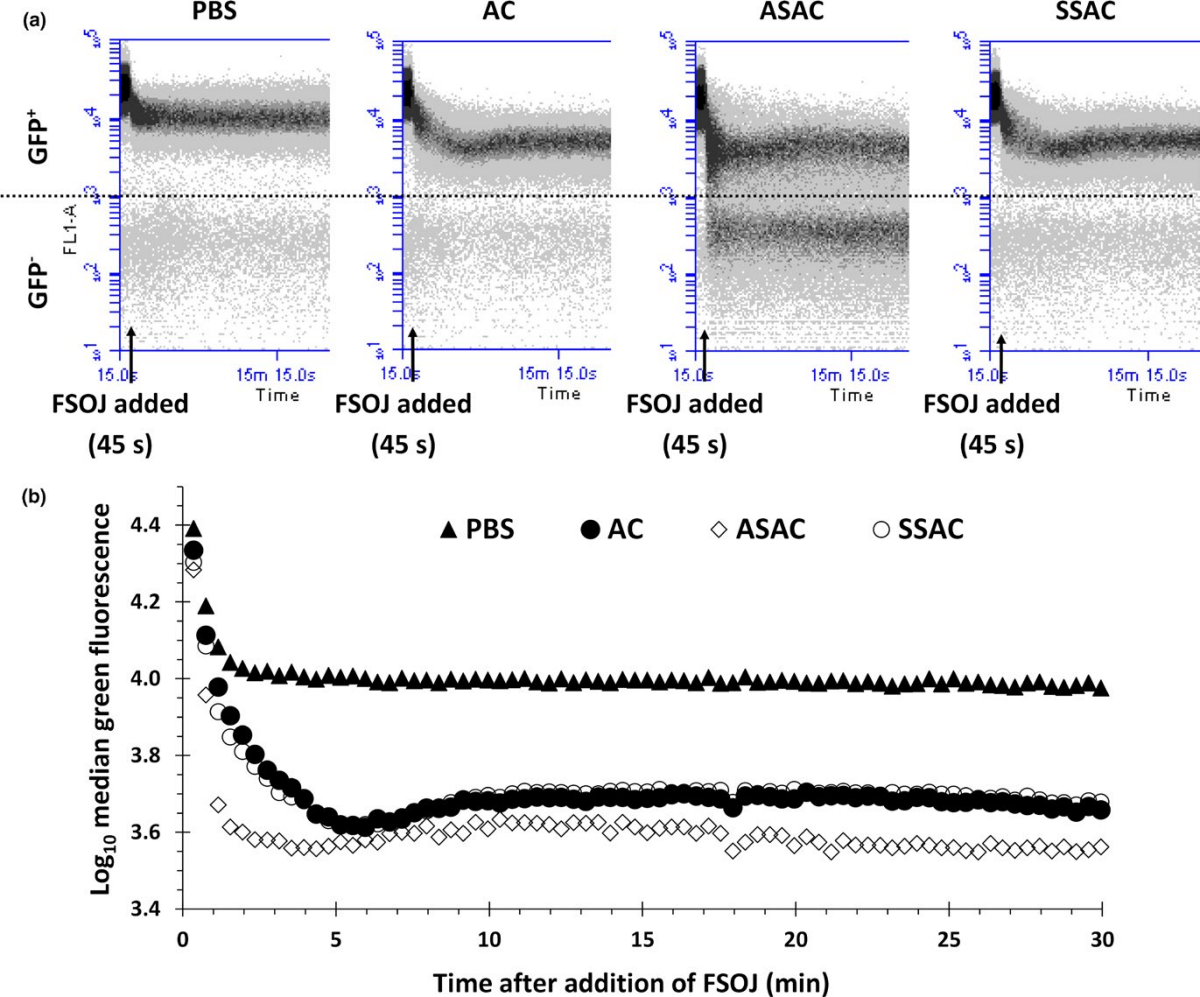
Real‐time flow cytometry study of the effect of available chlorine (AC)‐based sanitizers on the green fluorescence of *Escherichia coli* K‐12 SCC1 up to time 30 min postaddition of orange juice (OJ). (a) 2 × 10^10^ stationary‐phase cells were washed with 1 ml of Phosphate‐Buffered Saline (PBS) (control), 200 ppm AC, ASAC, or SSAC for 2 min. Cells were washed with PBS and diluted in fresh PBS to achieve a cell suspension with concentration of 2 × 10^7^ cells/ml. Then, 100 μl of the resultant cell suspension was placed in a tube and analyzed on the flow cytometer for 30 s (data collection started from time 15 s). After 45 s, 900 μl of OJ (filtered with 1.2 μm filter paper) was added to the cell suspension (indicated by arrow below the plot), gently mixed for 15 s, and analyzed for another 29 min. Here, only the data for the first 20 min of the experiment have been shown. (b) The median green fluorescence of the GFP ^+^ cells shown in the density plots above from time 0–30 min (postinoculation in OJ). Each point represents the median FI of the GFP ^+^ cells at 25 s intervals

Compared to PBS, washing cells with AC, ASAC, or SSAC solutions caused a significant reduction in the culturability of the cells preinoculation in OJ (Figure 4b). Upon addition of OJ, there was a marked decline in the culturability of the cells particularly in case of ASAC and SSAC samples (*p *<* *0.05). However, from day 1 onward, the culturability of the cells in OJ samples remained relatively constant in all samples.

### 3.4 Real‐time FCM‐based investigation of the cellular physiology

During the previous experiments, it was noted that the significant drop in the GFP^+^ population occurred very rapidly following addition of OJ, within the time taken to disperse bacteria in OJ (around 10 s). Therefore, in order to study the effects of AC‐based sanitizers on the viability of *E. coli* during this time period, it was decided to use real‐time FCM (Arnoldini, Heck, Blanco‐Fernández, & Hammes, 2013). Stationary‐phase bacteria were washed with either or PBS (control), 200 ppm AC, ASAC, or SSAC for 2 min, harvested by centrifugation, dispersed in fresh PBS, and analyzed on the flow cytometer for 30 s. While data were still being acquired, 900 μl of OJ (clarified and filtered with 1.2 μm filter paper) was added to the cell suspension and gently mixed for 15 s and analyzed for another 29 min. Figure 4a shows the density plots of the change in the green fluorescence intensity of the cells during the first 20 min of the study while Figure 4b shows the median green fluorescence of the GFP^+^ cells up to time 30 min after addition of OJ. With regard to the PBS‐washed cells, addition of OJ led to a 0.39 log reduction in the green fluorescence of GFP^+^ cells by 2 min postaddition. Nevertheless, after the initial reduction, the green fluorescence of GFP^+^ cells remained relatively constant throughout the experiment. Although green fluorescence of AC, ASAC, and SSAC‐washed cells was similar prior to addition of OJ, a significantly greater decrease in green fluorescence was observed following addition of OJ. Supplementation of the AC solution with Tween‐80 (SSAC) did not affect the green fluorescence of the GFP^+^ cells compared to cells washed with AC. However, acidification with 2% citric acid (ASAC) led to a larger GFP^−^ population and also a decrease in the green fluorescence of the GFP^+^ population. Unlike PBS‐washed cells, the initial reduction in green fluorescence of the GFP^+^ cells washed with 200 ppm AC (regardless of the supplementation with acid or surfactant) was followed by an increase in green fluorescence up to 10 min after addition of OJ. However, in the case of ASAC‐washed cells, this increase was followed by a gradual decrease in green fluorescence of the GFP^+^ cells during the remaining course of the study.

### 3.5 Simultaneous use of GFP, BOX and PI

The physiology of GFP^−^/PI^−^ cells was further investigated by the use of the lipophilic dye Bis‐(1,3‐Dibutylbarbituric Acid) Trimethine Oxonol (BOX) which enters cells with a collapsed membrane potential, thereby permitting identification of injured cells. As GFP and BOX both emit green fluorescence, exploratory experiments were performed. Figure S1 shows an example of the simultaneous use of GFP, BOX, and PI for determining the number of healthy, stressed, injured, and dead cells in a representative sample of OJ. FCM of unstained *E. coli* SCC1 cells revealed three populations on green fluorescence histograms (Figure S1a): A high fluorescent GFP^+^ population, (healthy cells); a medium‐fluorescence population, (stressed cells); and a low fluorescence, GFP^−^ population thought to comprise stressed, injured, and dead cells (the latter later being differentially stained with PI). This low‐fluorescence population was further characterized by staining with BOX (Figure S1b). GFP^−^ cells with a collapsed membrane potential became fluorescent; the low‐fluorescence population decreased from 7.6% to 3.3% indicating that 4.4% of GFP^−^ cells had no membrane potential, hence stained with BOX. Addition of BOX did not affect the green fluorescence of GFP^+^ cells. Finally, PI was added (Figure S1c), which stained 0.3% of cells red; this was the dead population. By subtracting the percentage of dead cells from the total percentage of GFP^−^ cells with no membrane potential (BOX^+^), it was possible to determine the percentage of injured cells (GFP^−^ BOX^+^ PI^−^; 4.1%).

### 3.6 Effect of organic acids

**Figure 5:**
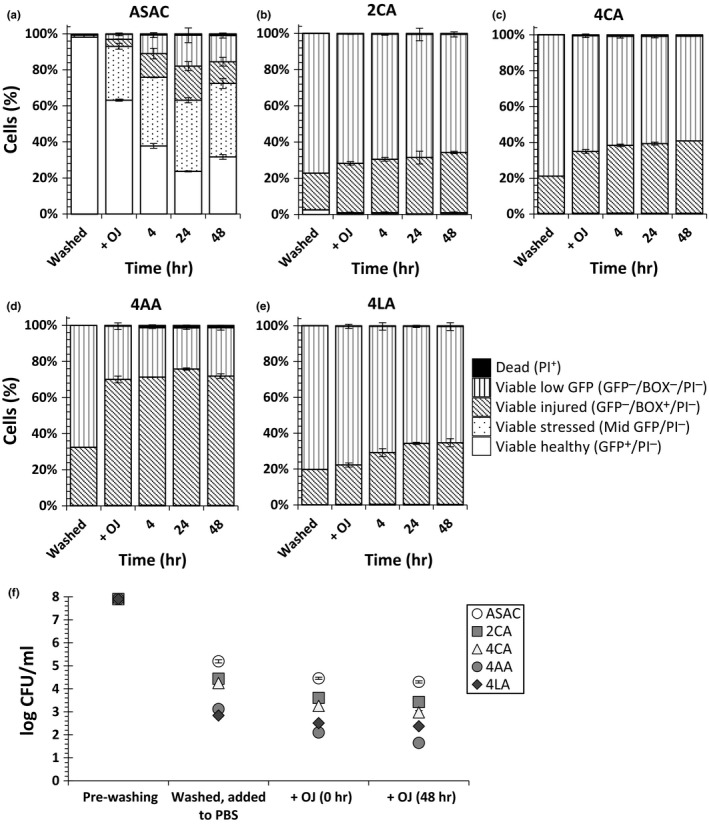
The effects of washing *Escherichia coli* K‐12 SCC1 with organic acid on its subsequent viability in orange juice (OJ) during 48‐hr incubation at 4°C compared to ASAC‐washed cells. The method used for this experiment was similar to that described in Figure 1, except cells were washed with either (a) ASAC (pH 6.0), (b) 2% citric acid (2CA, pH 2.1), (c) 4% citric acid (4CA, pH 1.9), (d) 4% lactic acid (4LA, pH 2.1), or (e) 4% acetic acid (4AA, pH 2.5). Samples were incubated at 4°C for 2 days. “Washed” and “+OJ” refer to the before and after addition of OJ at time 0 hr. Error bars are the ±*SD* of the mean value obtained at each time point for each type of population (e.g., healthy GFP ^+^, injured) and not the total percentage of the cells. (f) Culturable cells were determined by plate count pre‐ and postwashing and after addition of OJ (+OJ) and after 48 hr

As was shown above, washing the cells with ASAC had a significant effect on the subsequent stress response of the cells in OJ. However, these results raised the question of whether this effect was due to the combined antimicrobial effects of the citric acid and AC or simply due to lowering the pH of the alkaline AC solution. Therefore, it was decided to investigate the effects of 2% or 4% citric acid (2CA and 4CA respectively), 4% lactic acid (4LA), and 4% acetic acid (4AA) on numbers of viable and culturable cells pre‐ and postinoculation in OJ (Figure 5). For this part of the study, samples were stained with both BOX and PI as described above. Compared to ASAC solution, washing the cells with organic acids had a significantly greater adverse effects on the physiology of the cells before addition of OJ (Figure 5a–e). Washing the cells with ASAC had no significant effect on the percentage of healthy cells, and therefore, more than 98% of the cells were healthy (Figure 5a). In contrast, significantly greater number of injured cells and far lower numbers of healthy cells were observed when cells were washed with organic acids (Figure 5b–e; *p *<* *0.0001 for all).

Subsequent addition of OJ to ASAC‐washed cells led to a significant reduction in the number of healthy cells during the first 24 hr of the experiment, followed by a significant increase at time 48 hr (*p *<* *0.05) (Figure 5a). With regard to the organic acid‐washed cells, 4AA‐washed cells exhibited the least resistance to OJ immediately after addition with a 37% increase in the injured BOX^+^ population. Although organic acids appeared to exert a greater adverse effect on viability of the cells after washing and after addition of OJ, the total percentage of PI^−^ (viable) cells remained relatively constant during the course of the study, similar to that observed for ASAC‐washed cells. As with physiology determined by FCM, culturability was decreased more by organic acids than ASAC (Figure 5f). The reductions in plate counts at time 0 hr postwashing were significantly higher for cells washed with 2CA, 4CA (both *p *<* *0.05), 4LA, and 4AA (both *p *<* *0.01). 4AA was found to be more effective than other sanitizers in reducing the culturability of *E. coli* subsequent to addition of OJ.

## 4 DISCUSSION

### 4.1 Effects of available chlorine

Numerous studies have investigated the effectiveness of AC‐based sanitizers in reducing the microbial load on the surface of fruits; however, the results have been conflicting (Parish et al., 2003). In light of this, we decided to model washing using the methods in the present manuscript. It was difficult to compare the results presented here to those reported in the literature, primarily because of the differences in the experimental methods used, typically with whole fruits or other produce being washed, and differences in the temperature and pH of the sanitizing solutions as well as the duration of the treatment (Bagci & Temiz, 2011; Neo et al., 2013; Pao & Davis, 1999; Pao et al., 2000; Sapers et al., 1999). These parameters could significantly influence the antimicrobial efficacy of the AC. For instance, the reduction in pH from alkaline to neutral could change the equilibrium between OCl^−^ and HOCl resulting in greater concentration of the latter which has been shown to exhibit stronger antimicrobial effects (Fukuzaki, 2006; McGlynn, 2004). In addition, unlike the current study in which the cells were suspended in PBS and washed with AC before their inoculation in OJ, in these studies cells were artificially inoculated on the surface of the produce before being immersed in or sprayed with AC‐based solutions. Moreover, in some studies the terms AC, chlorine, hypochlorite, NaOCl, and bleach have been used interchangeably, making it difficult to determine the exact concentration of free chlorine used (Chung, Bang, & Drake, 2006; McGlynn, 2004; Narciso & Plotto, 2005).

Chung and coworkers reported that the increase in concentration of NaOCl from 50 to 200 ppm resulted in a greater decrease in the total microbial, coliform, and *E. coli* counts (Chung, Huang, Yu, Shen, & Chen, 2011). The observed level of decrease in the plate count of *E. coli* was within the range of 1–2 log reduction reported in the literature for antimicrobial efficacy of 200 ppm AC (Parish et al., 2003; Sapers, 2001). Washing the cells with 200 ppm AC also resulted in a significant increase in the number of VBNC cells. The induction of VBNC in AC‐stressed cells has previously been reported (Dukan, Levi, & Touati, 1997; Kolling & Matthews, 2001; Oliver, Dagher, & Linden, 2005; Singh et al., 1986).

### 4.2 Effects of hydrogen peroxide

Available chlorine within the permitted range (maximum 200 ppm) is generally not capable of reducing the microbial load on the surface of the fruit in excess of 2 log, and therefore, it does not ensure the safe elimination of food pathogens from the surface of fresh produce (Parish et al., 2003; Sapers, 2001). This is particularly the case in fresh produce such as orange fruit with porous and hydrophobic surfaces (e.g., due to the presence of wax and essential oils) which can potentially protect the bacteria against AC action by reducing the accessibility of the aqueous AC‐based sanitizer to the bacteria (Adams et al., 1989; Beuchat & Ryu, 1997; Martinez‐Gonzales et al., 2011). It has been suggested that washing solution containing up to 5% H_2_O_2_ could be used as a suitable alternative to AC (Sapers et al., 1999, 2002). Wider uses of H_2_O_2_ as a sanitizer are reviewed by Parish et al. (2003).

In common with the results observed for AC‐washed cells, increasing the concentration of H_2_O_2_ led to a greater decrease in the number of healthy GFP^+^ cells and the plate viable count. However, except for 5% H_2_O_2_, washing cells with 1% and 2.5% H_2_O_2_ were not as effective as 200 ppm AC in reducing the population of healthy cells or plate count. Some studies have reported no significant effect on the viability of *E. coli* population on the surface of apples when the concentration of H_2_O_2_ was increased from 1% to 2.5% or 5% (Sapers et al., 1999, 2002). Others found 1%–5% H_2_O_2_ to be as effective as 200 ppm AC in reducing the *E. coli* population (Sapers, 2001; Sapers, Miller, Jantschke, & Mattrazzo, 2000). The discrepancy between our results and those reported in the literature is believed to be due to combined effects of using a different strain of *E. coli*, incubation of the stationary‐phase cells at 4°C for 24 hr before H_2_O_2_ treatment as well as using different temperature for the H_2_O_2_ solution (50°C instead of 30°C used in the current study) by these researchers.

### 4.3 Effects of acidified and surfactant‐supplemented AC

It appeared that the initial acid stress encountered by bacteria treated with ASAC increased the capability of the survivors to resist the very low pH of OJ. Nevertheless, acid or surfactant supplementation of 200 ppm AC did not affect the overall number of viable cells as determined by FCM. Adams and his colleagues showed that the acidification of 100 ppm AC from pH 8.8 to 4 with H_2_SO_4_ or various organic acids could increase the efficacy of AC for removing the microbial load on the surface of the lettuce (Adams et al., 1989). Moreover, supplementation with Tween‐80 decreased the microbial load by 34% compared to unsupplemented AC (Adams et al., 1989). However, because no other studies have used FCM to investigate the effect of acid or surfactant supplementation of AC, it is difficult to discuss the present results in the context of other studies; future work could focus on the relationship between numbers of healthy, viable, and culturable cells upon exposure to AC in the presence of acid and surfactant and possible implications for food processing. No significant difference was observed between the total number of culturable cells postinoculation in OJ regardless of the supplementation with acid or surfactant.

### 4.4 Effects of organic acids

Although both the dissociated and undissociated forms of organic acids can exert antimicrobial effects against a wide range of microorganisms, the undissociated form has been shown to be more effective. According to the “weak acid preservative” theory, upon entry of the undissociated organic acids into the cell they dissociate, leading to generation of excess protons, hence reducing the pH_*i*_ of the cells. In order to prevent the adverse effects of low pH_*i*_ on enzymatic activity and nucleic acids, cells actively employ ATP in order to extrude the excess proton and this eventually leads to cell death (Davidson & Harrison, 2003; Lu, Breidt, Perez‐Diaz, & Osborne, 2011; Salmond, Kroll, & Booth, 1984). In order for the undissociated weak acid to lower the pH_*i*_ and exert antimicrobial effects, it needs to be either small (containing fewer than 3 carbons) or lipophilic in order to be able to diffuse through the plasma membrane (Stratford & Eklund, 2003). Citric acid is not only a large weak acid (six carbons) but also hydrophilic (partition coefficient or *P*_oct_ of log −0.172). Lactic acid on the other hand is a small organic acid (3 carbons) which can pass through the plasma membrane. However, because of its high hydrophilicity (*P*_oct_ log −0.62), its diffusion is very slow. Therefore, both acids are believed to exert antimicrobial effects by acting as an acidulant reducing the pH of the environment. On the other hand, despite being a hydrophilic acid (*P*_oct_ log −0.319), acetic acid can pass through the membrane in undissociated form due to its small size (3 carbons) and therefore reducing the pH_*i*_ and causing intracellular damage (Stratford & Eklund, 2003). The diffusion of undissociated acetic acid and to some extent lactic acid through the membrane could explain the greater antimicrobial efficacy of these acids against *E. coli* compared to hydrophilic citric acid.

## 5 CONCLUSIONS

This study demonstrated the successful application of FCM for monitoring the efficacy of orange fruit washing procedures. The results confirmed the hypothesis that subjection of *E. coli* to sanitizers (200 ppm AC and 1% H_2_O_2_) followed by OJ induces the VBNC state. Acidification or supplementation of AC with surfactant did not affect the total number of viable or culturable cells in OJ. Real‐time FCM analysis was demonstrated to permit detection of fluctuations in pH_*i*_ and demonstrated differences in the effects of AC, ASAC, and SSAC. Compared to AC, organic acids had significantly greater adverse effects on both the health and culturability of the cells. 4% acetic acid was found to be more effective than 4% citric or lactic acid in reducing the population of healthy (BOX^−^) cells. Taken together, the data highlight the need to analyze bacterial viability and physiology using nongrowth‐dependent methods in order to quantify VBNC cells.

## CONFLICT OF INTEREST

TWO and AA were paid speaker expenses by BD for speaking at BD Accuri users' events.

## ETHICAL STATEMENT

This study does not involve any human or animal testing.
