# Supplementary material for: Flow cytometry and growth‐based analysis of the effects of fruit sanitation on the physiology of Escherichia coli in orange juice
Source: Food Sci Nutr. 2019 Feb 7;7(3):1072–83. doi: 10.1002/fsn3.947 (PMC6418452; doi:10.1002/fsn3.947)
Supplement: Supplementary file 1 [file FSN3-7-1072-s001.docx]

***Supplemental Information for***

**Flow cytometry and growth-based analysis of the effects of fruit sanitation on the physiology of *Escherichia coli* in orange juice**

Amir HP Anvarian^1†^, Madeleine P Smith^1^, Tim W Overton^1,2^*

^1^ Bioengineering, School of Chemical Engineering, and ^2^ Institute of Microbiology & Infection, The University of Birmingham, Birmingham B15 2TT, UK

* Corresponding author: [t.w.overton@bham.ac.uk](mailto:t.w.overton@bham.ac.uk); Telephone: +44 121 4145306; ORCID 0000-0003-3050-2549

Email addresses: MPS - [M.SMITH.2@bham.ac.uk](mailto:M.SMITH.2@bham.ac.uk) ; AHPA - [AAnvarian@lincoln.ac.uk](mailto:AAnvarian@lincoln.ac.uk) .

^†^Current address: University of Lincoln, National Centre for Food Manufacturing, Holbeach Technology Park, Park Road, Holbeach, Lincolnshire PE12 7PT, UK

**Supplemental Figure S1**

**A**

**B**

**C**


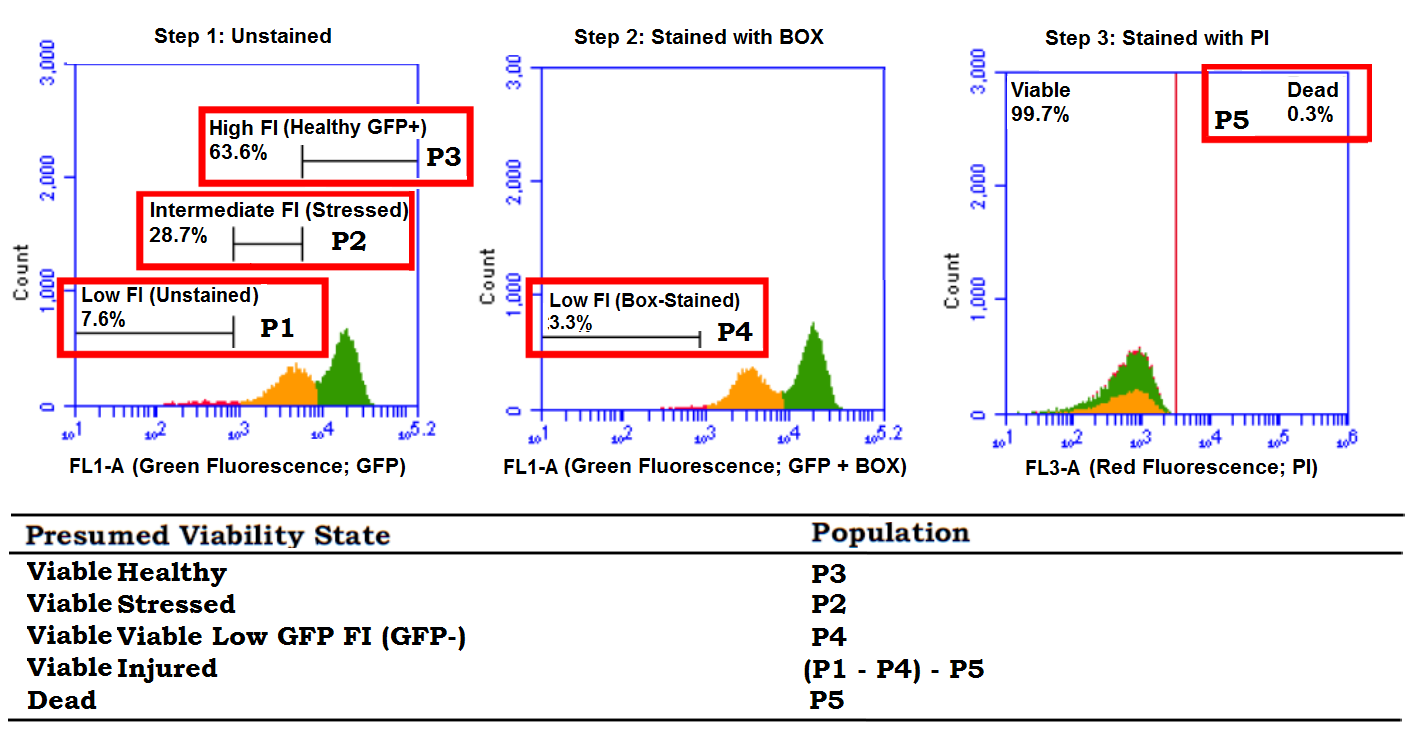


**Medium Fl. (Stressed)**

**Low Fl. (BOX^-^)**

**Low Fl. (Unstained)**

**High Fl. (Healthy)**

| **Physiological state** | **Fluorescence characteristics** | **Population above** |
| --- | --- | --- |
| Viable healthy | GFP^+^ PI^-^ | P3 |
| Viable stressed | Medium GFP | P2 |
| Viable injured | GFP^-^ BOX^+^ PI^-^ | (P1 - P4) - P5 |
| Viable low GFP | GFP^-^ BOX^-^ PI^-^ | P4 |
| Dead | PI^+^ | P5 |

**Supplemental Figure S1: Simultaneous use of two green fluorophores of GFP and BOX along with red fluorescent viability dye of PI in order to study the number of healthy, stressed, injured and dead *E. coli* K-12 SCC1 cells in OJ.** The method of experiment was similar to what was described in Figure 1, with the difference of using BOX in addition to PI. **(A)** Inoculation of cells in OJ resulted in an increase in the number of low GFP and/or GFP^−^ cells. Reduction in GFP fluorescence was presumed to be due to change in internal pH and subsequent denaturation of GFP. Therefore, GFP^+^ were considered healthy whereas low GFP cells were considered to be stressed or injured cells. **(B)** Samples were then stained with BOX in order to determine the percentage of injured cells without a membrane potential. Addition of BOX caused a reduction in the number of cells with low green fluorescence (compared to histogram A), indicating the staining of the injured GFP^−^ cells with BOX. **(C)** Staining the cells with PI showed the percentage of dead cells. Consequently, it was possible to calculate the percentage of injured cells by subtracting the number of dead cells (histogram C) and viable stressed GFP- cells (histogram B) from the pre- BOX staining percentage.
